# Detecting Cocircular Subsets of a Spherical Set of Points

**DOI:** 10.3390/jimaging8070184

**Published:** 2022-07-05

**Authors:** Basel Ibrahim, Nahum Kiryati

**Affiliations:** School of Electrical Engineering, Tel-Aviv University, Tel-Aviv 69978, Israel; baselibrahim@mail.tau.ac.il

**Keywords:** hough transform, spherical point set, circle detection, great circle, small circle

## Abstract

Given a spherical set of points, we consider the detection of cocircular subsets of the data. We distinguish great circles from small circles, and develop algorithms for detecting cocircularities of both types. The suggested approach is an extension of the Hough transform. We address the unique parameter-space quantization issues arising due to the spherical geometry, present quantization schemes, and evaluate the quantization-induced errors. We demonstrate the proposed algorithms by detecting cocircular cities and airports on Earth’s spherical surface. These results facilitate the detection of great and small circles in spherical images.

## 1. Introduction

Given a spherical set of points, consider the problem of detecting the largest subset of cocircular points, i.e., the largest subset of points lying on the same circle on the sphere. One brute-force solution, related to RANSAC [1], is to examine all possible triplets of points in the point set. Note that each triplet (if not colinear) defines a plane, the intersection of the plane with the sphere defines a circle, and each point in the triplet clearly lies on the circle. The point set can then be searched for additional points lying on that circle. This $O(N^{4})$ procedure, where *N* is the cardinality of the point set, is prohibitive for sizable point sets.

**Figure 1:**
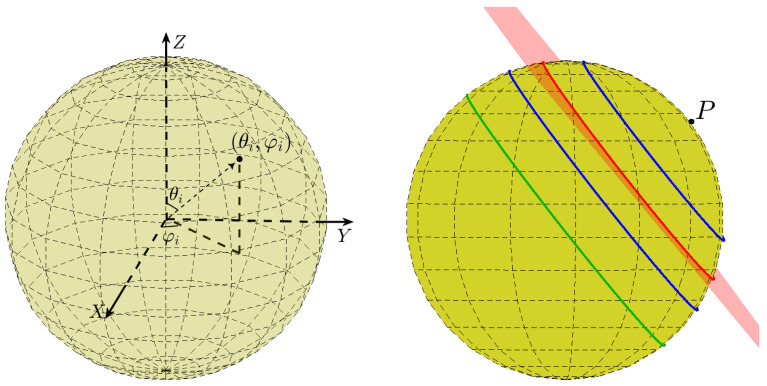
(**Left**) The angular coordinates $\theta_{i}$ and $\varphi_{i}$ of a point. (**Right**) The intersection of the sphere with a plane associated with the primary point *P* is a circle. The intersection of the sphere with each of the associated parallel planes is a different circle with a different radius. For example, the red plane of which the normal direction is determined by point *P* intersects the sphere at the red circle. The parallel plane that passes through the origin of the sphere is a great circle (green).

A cocircular subset of a spherical set of points is obviously coplanar. In special cases, the plane passes through the center of the sphere. In such cases, the intersection of the plane and the sphere is referred to as a *great circle*; see the green circle in Figure 1 (right). In the general case, when the plane does not pass through the center of the sphere, the intersection of the plane and the sphere is referred to as a *small circle*; see the blue and red circles in Figure 1 (right).

In the classical Hough transform [2], the problem of finding colinear points in the plane is replaced by the dual problem of finding concurrent lines or curves in a parameter space, often referred to as the Hough space. In practice, the parameter space is quantized and represented by an accumulator array, a voting process takes place, and finding the intersection of lines or curves in the parameter space is implemented as a search for the maximum. The Hough transform is fundamentally robust with respect to outliers, and has been formally associated with the M-estimation methodology for robust regression [3]. The Hough transform has also been related to conformal geometric algebra [4]. The time-complexity of the classical Hough transform is linear in the cardinality *N* of the dataset. Thus, when looking for the largest co-linear subset in a planar set of points, the Hough transform reduces the computational load from $O(N^{3})$ (the cost of checking each possible pair of points in the data; determining the line that connects the pair; and, for each other data-point, testing whether it lies on that line) to O(N).

The suggested approach to finding the largest subset of cocircular points in a spherical set of points is inspired by the Hough transform. Specifically, we characterize the circles (on the sphere) passing through a given point in the spherical set. The characterization is in the form of an equation constraining the parameters of the circles. We then search for the largest intersection of such equations, i.e., for the parameters specifying the circle passing through the largest possible number of points in the set.

As in the classical Hough transform, once the best-supported circle, i.e., the circle passing through the largest possible number of points, has been determined, finding the second-best-supported circle is straightforward. This can be reliably accomplished by identifying the data-points supporting the best circle, removing them from the dataset, and eliminating their contribution to the accumulator array (“*unvoting*”) [5,6,7]. Circle detection and unvoting can be iterated until all circles are found.

The precise function of the Hough transform is the detection of a geometric primitive, such as a straight line or circle, common to the largest possible subset of points in the dataset. Identifying the relevant points themselves can then be easily accomplished [5], but is not always necessary. Note that it is commonly said that the Hough transform detects geometric primitives in a digital image, but accomplishing this task in an actual image requires edge detection as a preprocessing step.

Images acquired using omnidirectional cameras can be represented as spherical images. Straight line segments in the scene are then transformed to great circle arcs in the spherical image. This has led to some interest in the detection of great circles in spherical images. With this motivation, Vasseur and Mouaddib [8] and Torii and Imiya [9] outlined a Hough algorithm and a randomized Hough algorithm, respectively, for great-circle detection on a sphere. These ideas have modern applications, such as road-line detection in driver-assistance systems [10].

In the detection of vanishing points, the search for convergence points in the image plane can be usefully replaced by the *intersection* of great circles on the Gaussian sphere [11]. This important task has been addressed using specialized Hough algorithms, leading to developments in spherical tessellation and discretization schemes [12,13] that are also useful in great-circle *detection* [14,15].

In geophysics, interest in great and small circle fitting arises in the context of volcano distribution analysis [16,17]. In these studies, specialized map projections transform great circles into an exact (gonomonic projection [17]) or approximate (UTM [16]) straight lines, allowing the use of the straight-line Hough transform [2] for great-circle detection. In the context of plate tectonics, Wessel [18] outlined the principles of a Hough transform for great-circle detection in the true spherical domain and sketched its generalization to small-circle detection. Wessel’s insightful concepts [18] have so far received surprisingly little attention.

In this paper, we present comprehensive solutions for the detection of cocircular subsets of a spherical set of points. We describe a geometric framework that supports both great- and small-circle detection and provide algorithms for both cases. We address the unique parameter-space quantization issues arising due to the spherical geometry, present quantization schemes, and evaluate the quantization-induced errors. We demonstrate the proposed algorithms by detecting cocircular cities and airports on Earth’s spherical surface.

## 2. Fundamentals

In this article, given a set of N>>1 co-spherical points, we present a way to detect the circle that passes through the largest possible number of points in the set. It is easy to show that the circle itself necessarily lies on the sphere.

A point in 3D space can be defined by its spherical coordinates, consisting of the radial distance *R* from the origin, the polar angle θ, and the azimuthal angle φ; see Figure 1 (left). Assuming co-spherical points, point *i* is determined by the angular coordinates $\theta_{i}$ and $\varphi_{i}$ alone.

Given an anchor point in 3D space and a normal direction, one can determine a plane that passes through the anchor point and fits the normal direction. By itself, a normal direction defines a set of parallel planes.

Viewing the angular coordinates of a (primary) point on the sphere as a normal direction therefore associates a set of parallel planes with that point. The intersection of each such plane with the sphere is a (primary) circle, as shown in Figure 1 (right).

**Figure 2:**
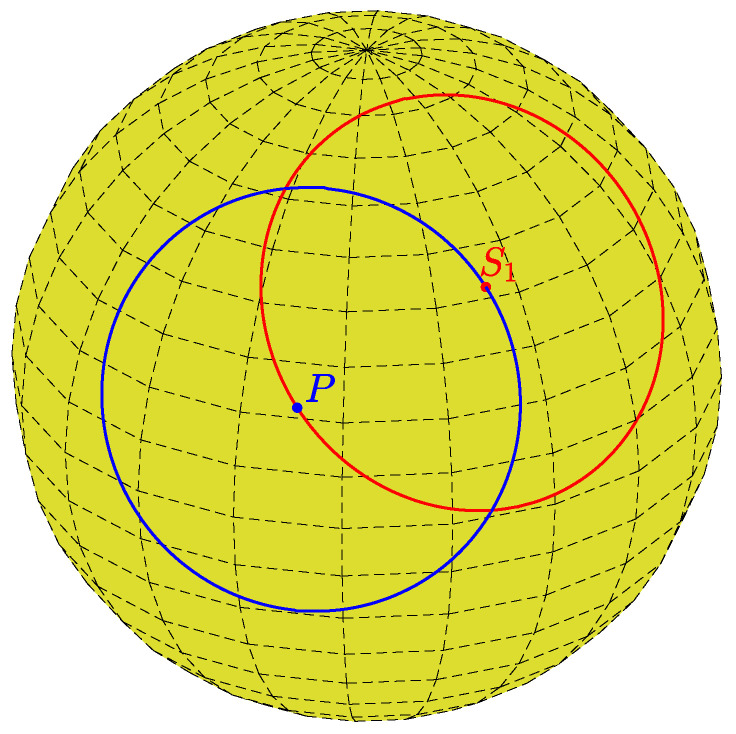
Each primary point (blue point *P*) defines a primary circle of radius *r* (blue). Each secondary point, such as the red point $S_{1}$ on the primary circle, defines a secondary circle of radius *r* (red) passing through the primary point.

Any (secondary) point on a primary circle of radius *r* (point $S_{1}$ in Figure 2), viewed as a normal direction, defines a secondary circle of radius *r* passing through the primary point (point *P* in Figure 2). Thus, given a primary point and a radius *r*, each secondary point is the center of a circle of radius *r* passing through the primary point.

**Figure 3:**
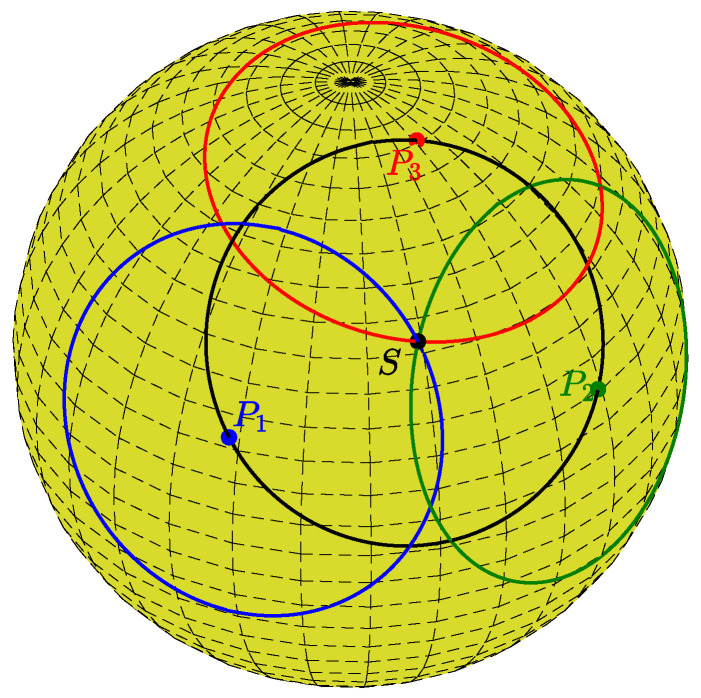
The red, green, and blue points $\left(P_{1},P_{2},P_{3}\right)$ are primary points located on the same circle (black). Each primary point defines a primary circle. All the primary circles intersect at a secondary point (black point *S*). That point defines a secondary circle (black) passing through the primary points.

Since this property holds for any secondary point on a primary circle, a set of primary circles for primary points located on the same circle will intersect at a specific secondary point, which is the center of the circle passing through all the primary points. This is illustrated in Figure 3, where the red, blue, and green points are primary points on the same (black) circle of radius *r*. The corresponding primary circles of radius *r* intersect at the black secondary dot *S*, which defines the secondary circle (black) passing through all the primary points.

Now, given a set of unit co-spherical points, i.e., a set of points $S=\{\left(\theta_{i},\varphi_{i}\right)\}$ located on the unit sphere, we look for the normal direction (θ,φ) that determines the circle of (pre-defined) radius *r* passing through the largest possible subset of points in *S*. The problem amounts to finding the largest co-circular (with given radius *r*) subset of *S*. We approach it by viewing each point $\left(\theta_{i},\varphi_{i}\right)$ as a primary point corresponding to a primary circle of radius *r*. We view (θ,φ) as a secondary point corresponding to a secondary circle on which a point $\left(\theta_{i},\varphi_{i}\right)$ lies. We select (θ,φ), compatible with the largest possible number of primary points $\left(\theta_{i},\varphi_{i}\right)$. In other words, we select (θ,φ), which is the largest intersection of primary circles.

As just presented, the problem and its solution strategy can be regarded as a generalization of the classical Hough transform for circles of known radius in the plane. In principle, one might indeed solve the problem by allocating a memory array on a spherical surface and incrementing memory cells on the primary circles corresponding to the data-set $S=\{\left(\theta_{i},\varphi_{i}\right)\}$ (voting). The memory cell with the largest accumulation will then correspond to (θ,φ), which is the largest intersection of primary circles, i.e., the normal direction defining the circle of radius *r* passing through the largest possible subset of points in *S*.

In practice, working with spherical memory arrays is highly inconvenient. We therefore carry out the computation using a planar memory array, where the orthogonal planar axes are θ and φ. Note, however, that the mapping of the conceptual spherical memory array to a planar array is non-trivial in both theory and practice, as is the drafting of planar maps of planet Earth [19]. As will be seen, this difficulty reveals itself in the context of the parameter-space quantization.

We can summarize these concepts as follows: Given a (unit) sphere and a radius *r*,

**Property** **1.** *A primary point $\left(\theta_{i},\varphi_{i}\right)$ on the sphere corresponds to a curve in the (θ,φ) parameter plane. The curve can be viewed as the mapping of the primary circle associated with the primary point from the sphere to the (θ,φ) plane.*

**Property** **2.** *A point in the (θ,φ) parameter plane corresponds to a secondary circle of radius r on the sphere.*

**Property** **3.** *Primary points lying on the same circle of radius r on the sphere correspond to curves through a common point in the (θ,φ) parameter plane.*

In the next section, we determine θ as a function of φ (or φ as a function of θ ). This is the voting pattern associated with a data point $(\theta_{i},\varphi_{i})\in S$.

## 3. The Voting Pattern

### 3.1. Derivation of the Voting Pattern

Consider a primary point $\left(X_{i},Y_{i},Z_{i}\right)$ on a unit sphere. We proceed to express the equation of the corresponding family of primary circles lying on parallel planes, as illustrated in Figure 1 (right). Generally, the equation of a plane in 3D space is of the form

(1) ax+by+cz=d

Note that (a,b,c) is the normal to the plane, and if it is normalized such that $a^{2}+b^{2}+c^{2}=1$, then *d* is the distance of the plane from the origin.

**Figure 4:**
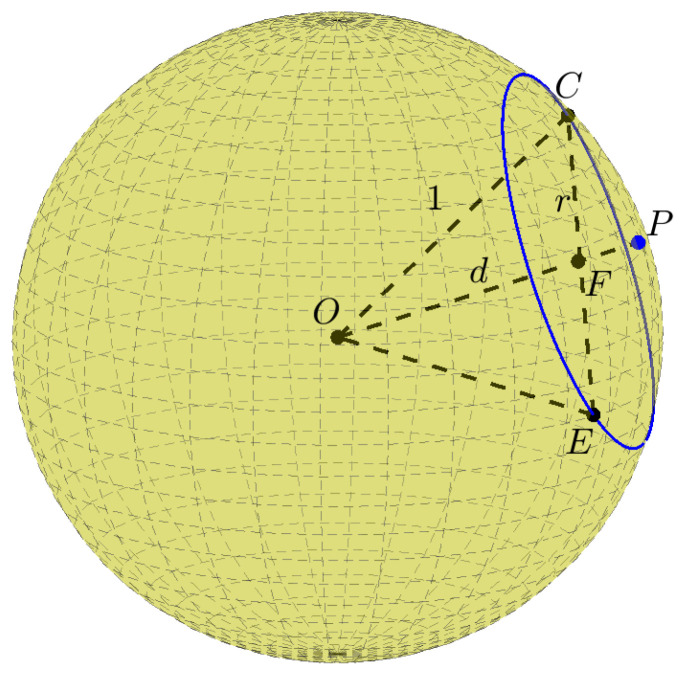
The relation between *d* and the circle radius *r*. P is a primary point on a unit sphere. The blue circle is the primary circle of radius *r* defined by *P*. The triangle CFO is right-angled.

Using Pythagoras’ theorem, for a unit sphere, the relation between *d* and the radius *r* of the circle is (see Figure 4):

(2) $$r=\sqrt{1-d^{2}}$$

In Equation (1), (a,b,c) is the normal to the plane. Here the normal is defined by the primary point. In terms of its angular coordinates $\left(\theta_{i},\varphi_{i}\right)$,

(3) $$\vec{n}=\left(\begin{array}{ccc} a & b & c \end{array}\right)=\left(\begin{array}{ccc} X_{i} & Y_{i} & Z_{i} \end{array}\right)=\left(\begin{array}{ccc} \sin\theta_{i}\cos\varphi_{i} & \sin\theta_{i}\sin\varphi_{i} & \cos\theta_{i} \end{array}\right)$$

Thus, the equation of the plane containing the primary circle is:

(4) $$\left(\begin{array}{ccc} \sin\theta_{i}\cos\varphi_{i} & \sin\theta_{i}\sin\varphi_{i} & \cos\theta_{i} \end{array}\right)\left(\begin{array}{c} x\\ y\\ z \end{array}\right)=d$$

The primary circle itself is the intersection of this plane with the unit sphere. In spherical coordinates, a point (x,y,z) on the unit sphere is

(5) $$\left(\begin{array}{c} x\\ y\\ z \end{array}\right)=\left(\begin{array}{c} \sin\theta \cos\varphi \\ \sin\theta \sin\varphi \\ \cos\theta \end{array}\right)$$

Substituting in the plane Equation (4) we obtain

(6) $$\left(\begin{array}{ccc} \sin\theta_{i}\cos\varphi_{i} & \sin\theta_{i}\sin\varphi_{i} & \cos\theta_{i} \end{array}\right)\left(\begin{array}{c} \sin\theta \cos\varphi \\ \sin\theta \sin\varphi \\ \cos\theta \end{array}\right)=d$$

Rearranging this, we obtain a relation between the angular coordinates θ,φ of secondary points on the primary circle.

$$\cos\theta \cos\theta_{i}+\sin\theta \cos\varphi \sin\theta_{i}\cos\varphi_{i}+\sin\theta \sin\varphi \sin\theta_{i}\sin\varphi_{i}=d$$

$$\cos\theta \cos\theta_{i}+\sin\theta \sin\theta_{i}\left(\cos\varphi \cos\varphi_{i}+\sin\varphi \sin\varphi_{i}\right)=d$$

$$\cos\theta \cos\theta_{i}+\sin\theta \sin\theta_{i}\cos\left(\varphi -\varphi_{i}\right)=d$$

(7) $$\cos\left(\varphi -\varphi_{i}\right)=\frac{d}{\sin\theta \sin\theta_{i}}-\cot\theta \cot\theta_{i}$$

(8) $$\begin{array}{c} \varphi^{I}=2\pi n+\cos^{-1}\left(\frac{d}{\sin\theta \sin\theta_{i}}-\cot\theta \cot\theta_{i}\right)+\varphi_{i}\\ \varphi^{II}=2\pi n-\cos^{-1}\left(\frac{d}{\sin\theta \sin\theta_{i}}-\cot\theta \cot\theta_{i}\right)+\varphi_{i} \end{array}\;\;\;n\in Z$$

**Figure 5:**
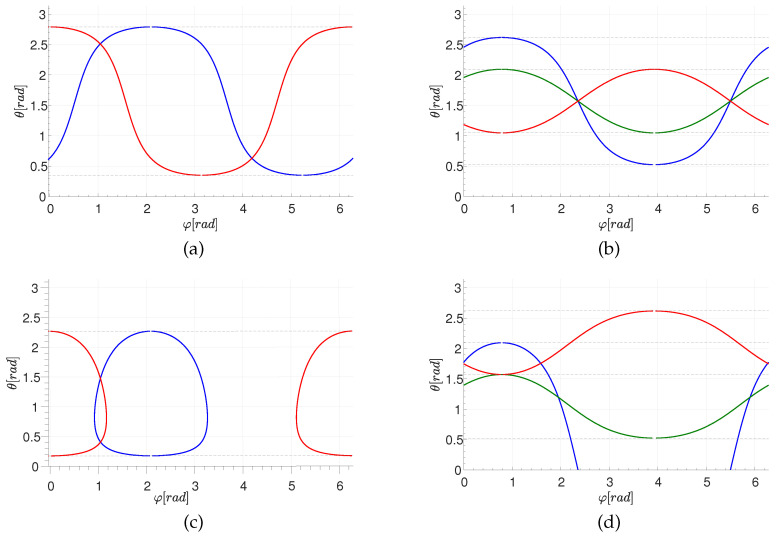

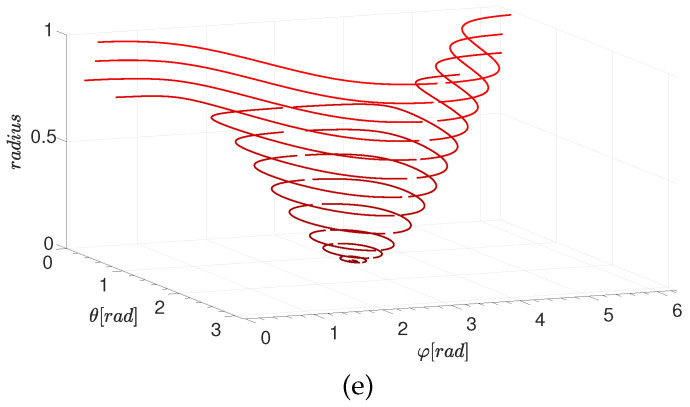
The locus of {θ,φ} pairs for selected values of $\theta_{i}$, $\varphi_{i}$, and *d*. (**a**,**b**) The case of d=0. (**a**): for two primary points with $\theta_{i}=70^{\circ }$; blue: $\varphi_{i}=120^{\circ }$, red: $\varphi_{i}=0^{\circ }$. (**b**): for three primary points with $\varphi_{i}=45^{\circ }$; blue: $\theta_{i}=60^{\circ }$, red: $\theta_{i}=30^{\circ }$, green: $\theta_{i}=150^{\circ }$. (**c**,**d**) Same as (**a**,**b**) but for d=0.5. (**e**) Illustrating the dependence on r=r(d). Here the primary point is $\varphi_{i}=180^{\circ },\theta_{i}=70^{\circ }$.

For a given primary point $\left(\theta_{i},\varphi_{i}\right)$ and a plane distance *d*, Equation (8) determines the azimuthal angle φ of a secondary point in terms of the polar angle θ. The two solutions reflect the fact that a circle of latitude (defined by θ) that intersects a primary circle (excluding the two circles of latitude that osculate the primary circle) intersects the primary circle at two points; see Figure 2 and Figure 3. The dependence on *n* is due to the periodicity of φ. Figure 5 shows the locus of {θ,φ} pairs for selected values of $\theta_{i}$, $\varphi_{i}$, and *d*.

As shown in Figure 5 (top), for the case of a great circle (d=0), we can express θ as a single-valued function of φ. Substituting d=0 in Equation (7),

(9) $$\cos\left(\varphi -\varphi_{i}\right)=-\cot\theta \cot\theta_{i}$$

Solving for θ,

(10) $$\theta =\pi n-\cot^{-1}\left(\cos\left(\varphi -\varphi_{i}\right)\tan\theta_{i}\right)\;n\in Z$$

### 3.2. Domain and Range of the Voting Pattern

We have so far seen that for a given primary point $\left(\theta_{i},\varphi_{i}\right)$, the φ coordinate of a secondary point (θ,φ) is obtained by substituting the θ coordinate in Equation (8). In this subsection, we proceed to show the dependence of the domain and the range of φ as a function of θ, as represented by Equation (8), on the primary point coordinates $\left(\theta_{i},\varphi_{i}\right)$ and the distance *d*.

As shown in Figure 4, for a given distance d>0, each (spherical) circle of radius *r* (Equation (2)) on a unit sphere is uniquely defined by one (spherical) point on the unit sphere. Any such spherical point can be represented by the spherical coordinates θ∈[0,π) and φ∈[0,2π). Therefore, any spherical circle with radius r<1 is uniquely defined by θ∈[0,π) and φ∈[0,2π).

**Figure 6:**
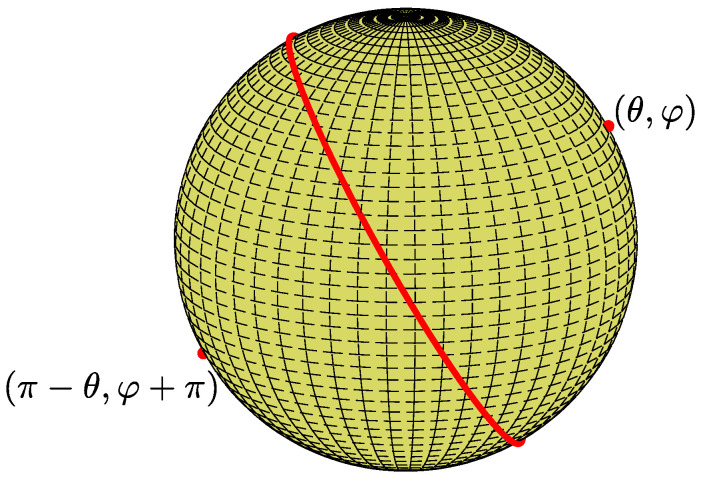
In the case of the great circles, d=0, two antipodal spherical points (red), one at each hemisphere, define the same great circle.

In the case of great circles, d=0, the points (θ,φ) and (π−θ,π+φ) (red points in Figure 6) define the same plane. In other words, each great circle (red) is represented by two spherical points, one at each hemisphere. Consider the subset of spherical points in the range θ∈[0,π) and φ∈[0,π). There is a bijective transformation from this subset to the set of great circles on the unit sphere. To conclude, each spherical point with θ∈[0,π) and φ∈[0,π) uniquely defines a great circle.

Returning to the general case, for a given primary point $\left(\theta_{i},\varphi_{i}\right)$, the spherical points (θ,φ) that satisfy Equation (8) represent the secondary circles on which the primary point lies. We are interested in secondary points with φ∈[0,2π), so *n* is selected such that $\varphi^{I},\varphi^{II}\in \left[0,2\pi \right)$.

Given a primary point and *d*, the domain of Equation (8) consists of θ coordinates of the secondary points. The θ value of the secondary points are such that the argument of the inverse cosine function satisfies

$$\frac{d}{\sin\theta \sin\theta_{i}}-\cot\theta \cot\theta_{i}\in \left[-1,1\right]$$

$$|\frac{d}{\sin\theta \sin\theta_{i}}-\cot\theta \cot\theta_{i}|\leq 1$$

(11) $$\left|\frac{d-\cos\theta \cos\theta_{i}}{\sin\theta \sin\theta_{i}}\right|\leq 1$$

Denote by $\theta_{lim}^{I,II}$ the limiting values of θ, i.e., the values corresponding to the two circles of latitude osculating the primary circle:

$$\frac{d-\cos\theta_{lim}^{I,II}\cos\theta_{i}}{\sin\theta_{lim}^{I,II}\sin\theta_{i}}=\pm 1$$

$$d-\cos\theta_{lim}^{I,II}\cos\theta_{i}=\pm \sin\theta_{lim}^{I,II}\sin\theta_{i}$$

$$\cos\left(\theta_{lim}^{I,II}\mp \theta_{i}\right)=d$$

(12) $$\theta_{lim}^{I,II}=2\pi n\pm \cos^{-1}\left(d\right)\pm \theta_{i}$$

We choose *n* such that $\theta_{lim}^{I,II}\in \left[0,\pi \right)$. Therefore, given a primary point and a distance *d*, the θ coordinate of any point on the secondary circle lies between $\theta_{lim}^{I}\in \left[0,\pi \right)$ and $\theta_{lim}^{II}\in \left[0,\pi \right)$. Note that the largest difference between $\theta_{i}$ (of the primary point) and θ of a secondary point is obtained for any of the limiting values $\theta =\theta_{lim}^{I,II}$. The difference is the angle between the radius vectors pointing to the primary point and to either of the two points of osculation, and this angle is equal to $\cos^{-1}d$.

### 3.3. Sphere of Radius R

The result expressed by Equations (8) and (10) can be easily generalized from the unit sphere to a sphere of radius *R*. The vector representing a point on the sphere (Equation (5)) is multiplied by *R*. Substituting in Equation (4), we obtain

$$R(\cos\theta \cos\theta_{i}+\sin\theta \sin\theta_{i}\cos\left(\varphi -\varphi_{i}\right))=d$$

where, generalizing Equation (2), *d* is now related to the radius of the circle by $d=\sqrt{R^{2}-r^{2}}$. Thus, Equations (6) and (8) will hold if *d* is replaced by $d_{R}$, where

$$d_{R}=\frac{d}{R}=\frac{\sqrt{R^{2}-r^{2}}}{R}=\sqrt{1-{\left(\frac{r}{R}\right)}^{2}}$$

So, technically, an algorithm to search for circles of radius *r* on a unit sphere can be readily applied to spheres of radius *R* by normalizing the circle radius *r* by *R*.

## 4. Algorithm for Great-Circle Detection

In this section, we present an algorithm for finding great circles on a sphere. More accurately, given a set $S=\{\left(\theta_{i},\varphi_{i}\right)\}$ of *N* co-spherical points, we find the great circle that passes through the largest subset of *S*. Specifically, the (secondary) great circle passing through the largest possible subset of (primary) points is defined by the (secondary) point $\left(\varphi_{max},\theta_{max}\right)$. The algorithm belongs to the Hough transform family, since it is based on voting in a quantized parameter space.

The parameter plane is (φ,θ) is limited to the rectangle φ∈[0,π) θ∈[0,π) and tessellated by rectangular cells. Note that, in the case of great circles, Equation (10) specifies θ as a function of φ, hence the parameterization is expressed as (φ,θ). In the general case, where Equation (8) determines φ as a function of θ, the parametrization will be written as (θ,φ). Each cell represents a subset of (secondary) spherical points, and is of the form $\varphi \in [\tilde{\varphi }-\frac{\Delta \varphi }{2},\tilde{\varphi }+\frac{\Delta \varphi }{2}]$ and $\theta \in [\tilde{\theta }-\frac{\Delta \theta }{2},\tilde{\theta }+\frac{\Delta \theta }{2}]$, where $\left(\tilde{\varphi },\tilde{\theta }\right)$ is the center of the cell. In common Hough algorithms, the cells are of uniform shape and size, their centers form a set of rectangular grid points, and each cell is represented by an accumulator M(i,j) in a memory array, referred to as the accumulator array.

For each (primary) point in the data-set, we compute θ as a function of φ (Equation (10)) and increment the accumulators M(i,j) corresponding to cells along the θ(φ) curve. The accumulation stage of the algorithm can be visualized as drawing the discretized curves θ(φ) in the (φ,θ) parameter plane for all primary points. Eventually, the indices $\left(i_{max},j_{max}\right)$ of the accumulator with the maximal reading represent the parameters $\left(\varphi_{max},\theta_{max}\right)$ of the great circle. Rather than determining all the cells that the curve θ(φ) passes through, the common Hough transform practice is to approximate the process by sampling the argument (φ) and quantizing the function (θ). This leads in our case to Algorithm 1:
**Algorithm 1:** great-circle detectionInitialize the matrix (accumulator array) *M* with zeros. For each primary point 1 to *N*        For each φ∈[0,π) step Δφ               Calculate θ(φ) by Equation (10).               Round each θ(φ) to the nearest integer multiple of Δθ.               Increment the accumulator of *M* corresponding to (φ,θ). Find $\varphi_{max}$ and $\theta_{max}$ corresponding to the entry with the largest accumulation in *M*.

The computational complexity of the algorithm is linear in the number of primary points *N*. With rectangular tessellation of the parameter plane, the computational load also depends on the number of discrete φ values, $N_{\varphi }=\pi /\Delta \varphi$, and on the number of discrete θ values, $N_{\theta }=\pi /\Delta \theta$. Specifically, the computational complexity of the voting stage is $O(N\cdot N_{\varphi })$ and that of the search stage is $O(N_{\varphi }\cdot N_{\theta })$.

Note that the approximation, sampling θ and quantizing φ rather than determining the cells that the curve θ(φ) passes through, leads to a pitfall. Since the magnitude of the slope of θ(φ) can be large, the discretized θ(φ) curves may not be digitally connected [20]. Thus, they may not intersect in the digital domain even though they do intersect in the continuous domain. This hazard may not be apparent in cases where *N* is large and intersections are dense, but may manifest itself in cases where *N* is small. This may then lead to peak-spreading in the parameter plane, and consequently to misdetection and poor localization of peaks. In contrast, if the approximation is discarded and the cells intersected by the curve are incremented, the discretization of the curve amounts to the classical square quantization scheme [21], and the discretized curve is digitally connected.

## 5. Algorithm for General (Radius *r*) Circle Detection on a Sphere

We now consider the more general problem—detecting circles of any given radius *r* on the sphere. Here, the given circle radius may not coincide with the radius of the sphere; hence, the circles to be detected are not necessarily great circles. Formally, given a set $S=\{\left(\theta_{i},\varphi_{i}\right)\}$ of co-spherical points and a circle radius *r*, we find the circle of radius *r* passing through the largest subset of *S*. In this case, the parameter plane is limited to a larger rectangle θ∈[0,π) φ∈[0,2π). We compute $\varphi^{I}$ and $\varphi^{II}$ as a function of θ for each primary point using Equation (8), and increment the accumulators corresponding to cells intersected by the $\varphi^{I,II}\left(\theta \right)$ curves. Approximating the process by sampling the argument θ and quantizing the functions $\varphi^{I,II}$ leads to Algorithm 2:
**Algorithm 2:** General (any given radius *r*) circle detection on a sphereInitialize the matrix (accumulator array) *M* with zeros.Normalize the radius *r* by the sphere radius *R*.Calculate *d* by Equation (2).For each primary point 1 to *N*      For each θ∈[0,π) step Δθ            If θ satisfies Equation (11) then                  Calculate $\varphi^{I}\left(\theta \right)$ and $\varphi^{II}\left(\theta \right)$ by Equation (8).                  Round $\varphi^{I,II}\left(\theta \right)\in \left[0,2\pi \right)$ to the nearest integer multiples of Δφ.                  Increment the accumulators of *M* corresponding to $\left(\theta ,\varphi^{I,II}\right)$.Find $\theta_{max}$ and $\varphi_{max}$ corresponding to the entry with the largest accumulation in *M*.

Here the computational complexity of the voting stage is $O(N\cdot N_{\theta })$ and that of the search stage is again $O(N_{\theta }\cdot N_{\varphi })$, where in this case $N_{\theta }=2\pi /\Delta \theta$. The accumulation stage of Algorithm 2 can be visualized as drawing the discretized $\varphi^{I,II}\left(\theta \right)$ curves in the (θ,φ) parameter plane for all primary points. As discussed in Section 4, care should again be taken when considering the use of the voting approximation (sampling the argument θ and quantizing the functions $\varphi^{I,II}$ rather than determining the cells that the curves pass through).

## 6. Parameter-Space Quantization and Error Analysis

### 6.1. Preliminaries and Motivation

In Hough transform algorithms [2], a data (image) point is mapped into a space of parameters for a class of geometric primitives (such as straight lines). The parameter space is quantized into cells and represented by an array of accumulators. The mapping is carried out by incrementation of the relevant accumulators (voting). Once all data points have voted, a search for the maximum in the accumulator array reveals the parameters of the geometric primitive instance that are best supported by the data.

Parameter space quantization is an essential element of the Hough transform. On the one hand, it determines the resolution of the output parameters. On the other hand, it reflects the tolerance of the algorithm to location errors in the data. Thus, coarse quantization leads to poor output resolution, but quantization that is too fine may lead, in the presence of data location errors, to peak-spreading in the accumulator array and consequent detection errors.

In the Hough transform for straight lines using the normal parameterization, uniform quantization of the (ρ,θ) parameter space into cells of equal size implies an equal (translation- and rotation-invariant) measure of the infinite set of straight lines represented by each cell [2,22,23]. In the proposed algorithms for great- and small-circle detection on a sphere, the parameter space is (in principle) an isomorphic spherical parameter space. This is analogous to the isomorphism between the image space and the parameter space in the Hough transform for circles of known radius [2]. Note that the area of a cell in the spherical parameter space is, due to rotational symmetry, a rotation-invariant measure of the infinite set of the spherical circles represented by the cell. We therefore wish to quantizate the spherical parameter space into cells of similar area.

### 6.2. Towards Uniform Spherical Quantization

Working with spherical memory arrays is currently impractical. We therefore carry out the computation using a planar memory array, where the orthogonal planar axes are θ and φ. Nevertheless, mapping the conceptual spherical array to a planar array is problematic [19]. Specifically, in the context of parameter-space quantization, uniform (rectangular) quantization in the plane, via uniform quantization of the individual angular coordinates θ and φ, corresponds to cells of non-uniform area on the sphere. Assuming small quantization steps Δθ and Δφ in the plane, the spherical surface area of the corresponding cell at θ and φ is approximately

(13) ΔS≈ΔθΔφsinθ

where ΔS is largest at the equator, where θ=π/2, and equal to ΔθΔφ. For non unit spheres, ΔS is scaled by $R^{2}$ where *R* is the radius.

**Figure 7:**
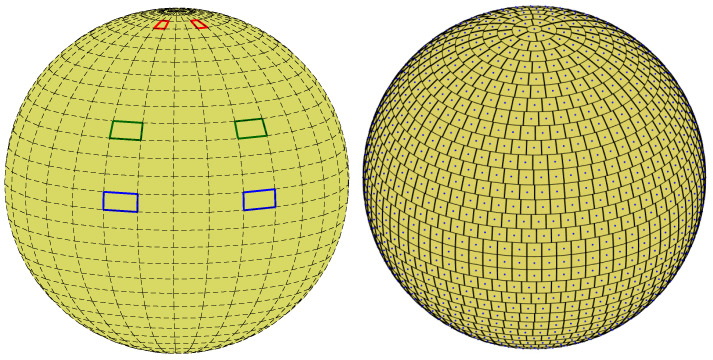
(**Left**) Uniform rectangular quantization of the parameter plane, i.e., uniform quantization of the angular coordinates θ and φ, corresponds to cells of non-uniform area on the sphere. Cells are small near the poles and large near the equator. (**Right**) Almost-equal-sized cell quantization.

Cells of non-uniform area imply biased voting. One way to handle bias is by normalizing each accumulator count by the area of the corresponding cell. Note, however, that the normalization is associated with singularity at the poles. Lutton et al. [13] suggested using *almost-equal-sized cell quantization*, letting Δφ depend on θ; see Figure 7. In our case, almost-equal-sized cell quantization is obtained by letting

(14) $$\Delta \varphi^{n}=\frac{\Delta S}{\Delta \theta \sin\theta_{n}}$$

where the layer number *n* is obtained by rounding θ/Δθ to the nearest integer, $\theta_{n}=n\Delta \theta$, and ΔS is the almost uniform cell size (spherical surface area).

**Figure 8:**
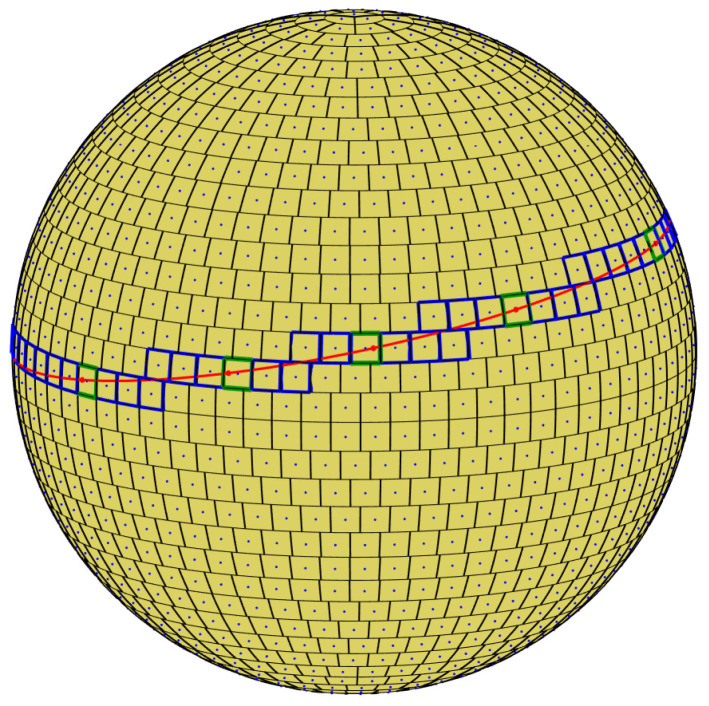
Incrementing all cells through which the continuous voting curve passes means voting for both the blue and green cells. In contrast, sampling θ and quantizing φ generates the centers of the green cells alone.

When voting into the (non-uniform) planar array corresponding to almost-equal-sized cells on the sphere, we again need to decide whether to increment all cells through which the continuous voting curve passes, or to employ the approximation—sampling θ and quantizing φ in the context of general circles. The two options are illustrated in Figure 8. Incrementing all cells through which the continuous voting curve passes means voting for both the blue and green cells. In contrast, sampling θ and quantizing φ generates (the centers of) the green cells alone.

**Figure 9:**
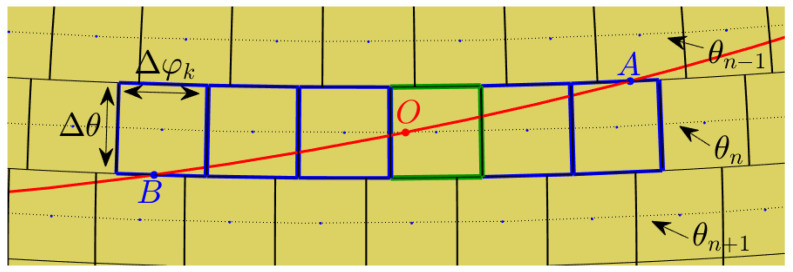
At layer $\theta_{n}$ we increment the accumulator(s) between *A* and *B* inclusively. On the sphere, these points are the intersections of the primary circle with the upper and lower boundaries of the $\theta_{n}$ layer. In the respective planar array, the points *A* and *B* are the intersections of the voting curve corresponding to the primary circle with the $\theta_{n}$ layer. Had the approximation been employed, only the green cell would have been incremented, as its center is the closest (in terms of φ) to the intersection of the curve with the sampled value of θ.

In our analysis and experiments, the first option is followed, i.e., the approximation is not employed and all cells through which the continuous voting curve passes are incremented. Technically, referring to Figure 9, at layer $\theta_{n}$ we increment the accumulator(s) between *A* and *B* inclusively.

### 6.3. The Distance of Primary Points from the Common Secondary Circle

Consider the cell centered at $\left(\theta_{max},\varphi_{max}\right)$ that received the largest number of votes from primary points. In other words, the cell centered at $\left(\theta_{max},\varphi_{max}\right)$ is the one intersected by the largest number of primary circles centered at primary points. Due to primary points’ location errors, and due to the finite (i.e., not infinitesimal) cell size, the primary circles intersecting the cell do not usually intersect at a single point within the cell themselves. Thus, typically, the primary points that have voted for the cell are only roughly co-circular. The common secondary circle passing near these primary points is defined by the approximate secondary point $\left(\theta_{max},\varphi_{max}\right)$, i.e., by the center of the cell.

**Figure 10:**
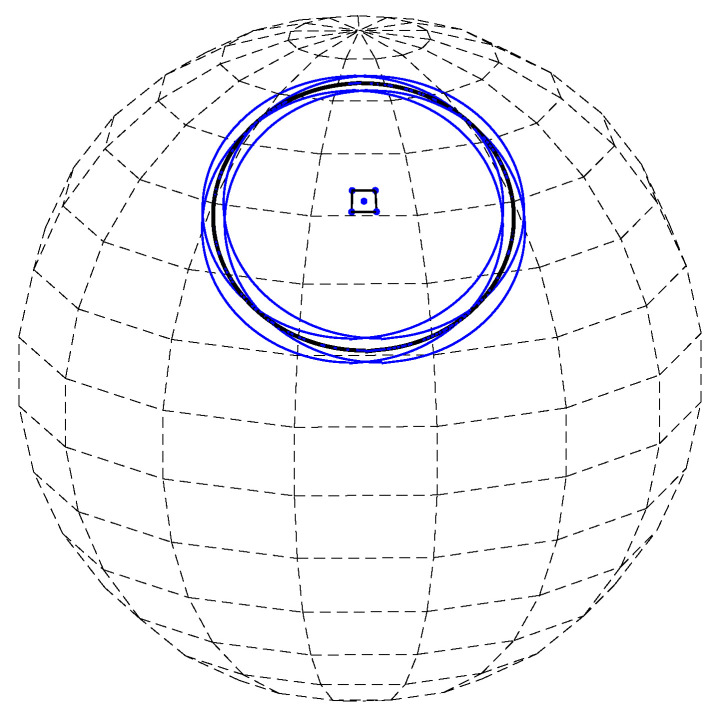
A cell centered at $\left(\theta_{max},\varphi_{max}\right)$. The black circle is the common secondary circle defined by the center of this cell. The four blue circles are the circles defined by the corners of the cell.

What is the maximum distance of the primary points contributing to the cell from the secondary circle defined by $\left(\theta_{max},\varphi_{max}\right)$? Furthermore, how does this depend on the finite cell size? This distance determines, on the one hand, the resolution of the algorithm and, on the other hand, its tolerance to noise (errors) in the location of the data (primary) points. To answer these questions, we calculate the distance between the common secondary circle (the black circle in Figure 10) and the circles defined by the four corners of the cell. The coordinates of the four corners are

$$\begin{array}{c} \left(\theta_{max}+\frac{\Delta \theta }{2},\varphi_{max}+\frac{\Delta \varphi^{k}}{2}\right)\\ \left(\theta_{max}+\frac{\Delta \theta }{2},\varphi_{max}-\frac{\Delta \varphi^{k}}{2}\right)\\ \left(\theta_{max}-\frac{\Delta \theta }{2},\varphi_{max}+\frac{\Delta \varphi^{k}}{2}\right)\\ \left(\theta_{max}-\frac{\Delta \theta }{2},\varphi_{max}-\frac{\Delta \varphi^{k}}{2}\right) \end{array}$$

where $\Delta \varphi^{k}$ is the layer-dependent quantization step (Equation (14)) at $\theta_{k}=\theta_{max}$, and the corresponding circles are shown blue in Figure 10.

**Figure 11:**
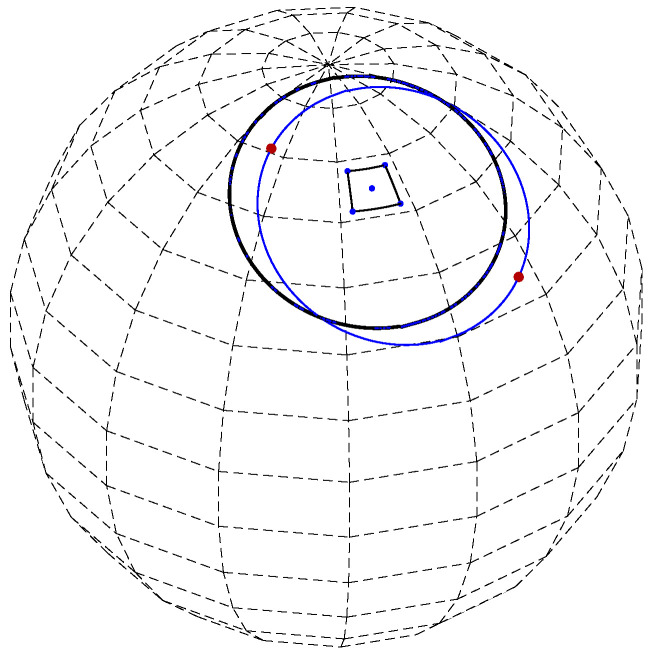
A secondary circle (black) and a corner circle (blue). The maximum distance of a point on the corner circle to the secondary circle is achieved by the two critical points (red).

Given one of the (corner) circles defined by a corner point (the blue circle in Figure 11), the largest distance from the common secondary circle is achieved by two *critical* points (red points in Figure 11) on the corner circle. One critical point is closer than the common secondary circle to the cell center, whereas the other critical point is more distant than the common secondary circle from the cell center.

**Figure 12:**
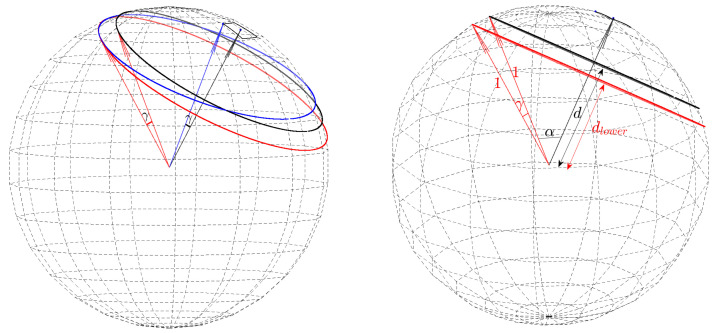
(**Left**) The angle γ between the (red) radius vector pointing at a critical point and its respective closest point on the common secondary circle (black) is equal to the angle between the radius vector (black) corresponding to the secondary point at the center of the cell $\left(\theta_{max},\varphi_{max}\right)$ and the radius vector (blue) associated with a corner point. (**Right**) The common secondary circle (black) and one of the critical circles (red). The spherical radius vector defined by the angular coordinates $\left(\theta_{max},\varphi_{max}\right)$ of the secondary point passes through the centers of the common secondary circle and the two critical circles, and this radius vector is perpendicular to the planes containing the circles. *d* and $d_{lower}$ are the distances of the common secondary circle and the (lower) critical circle from the origin of the sphere, respectively.

Consider any of the two critical points and its respective closest point on the secondary circle. Viewing the critical point and its closest point as (spherical) radius vectors, let γ denote the angle between them. Let *a* denote the radius vector corresponding to the secondary point $\left(\theta_{max},\varphi_{max}\right)$, and let *b* denote the radius vector associated with the corner point. Referring to Figure 12 (left), we observe that, since *a* is normal to the secondary circle and *b* is normal to the corner circle, γ is also the angle between *a* and *b*. Assuming a unit sphere,

(15) $$\gamma =\cos^{-1}\left(a\cdot b\right)$$

The angle γ can be viewed as an angular distance measure between the cell center $\left(\theta_{max},\varphi_{max}\right)$ and one of its corners. With almost-equal-sized cell quantization, γ is similar between cells and between corner points of a particular cell.

**Figure 13:**
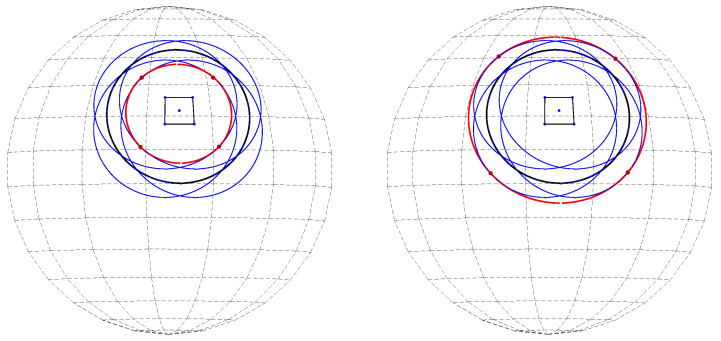
(**Left**) The four critical points (red) closer to the cell center and the (higher) critical circle they define. (**Right**) The four critical points (red) more distant from the cell center and the (lower) critical circle they define.

Cells have four corner points. Each corner point is associated with two critical points, one closer to and one more distant from the cell center. The eight critical points can be divided into two subsets, each with four critical points. One subset contains the four critical points, shown red in Figure 13 (left), closer to the cell center, whereas the other subset contains the four critical points more distant from the cell center, shown in red in Figure 13 (right). The angular distance γ between any of the eight critical points and its closest point on the common secondary circle is similar. This means that the four critical points in each of the two subsets are nearly co-circular. The two circles, shown in red in Figure 13, each defined by the four critical points in each of the two subsets, are referred to as *lower and higher critical circles*. They are on planes parallel to each other and also to the secondary circle plane, where the lower critical circle is closer to the origin and the higher is more distant from the origin. Specifically, their distances from the origin are

(16) $$d_{lower}=\cos\left(\cos^{-1}d+\gamma \right)\;d_{higher}=\cos\left(\cos^{-1}d-\gamma \right)$$

The spherical radius vector defined by the angular coordinates $\left(\theta_{max},\varphi_{max}\right)$ of the secondary point passes through the centers of the secondary circle and the two critical circles, and is perpendicular to (the planes containing) them; see Figure 12 (right).

**Figure 14:**
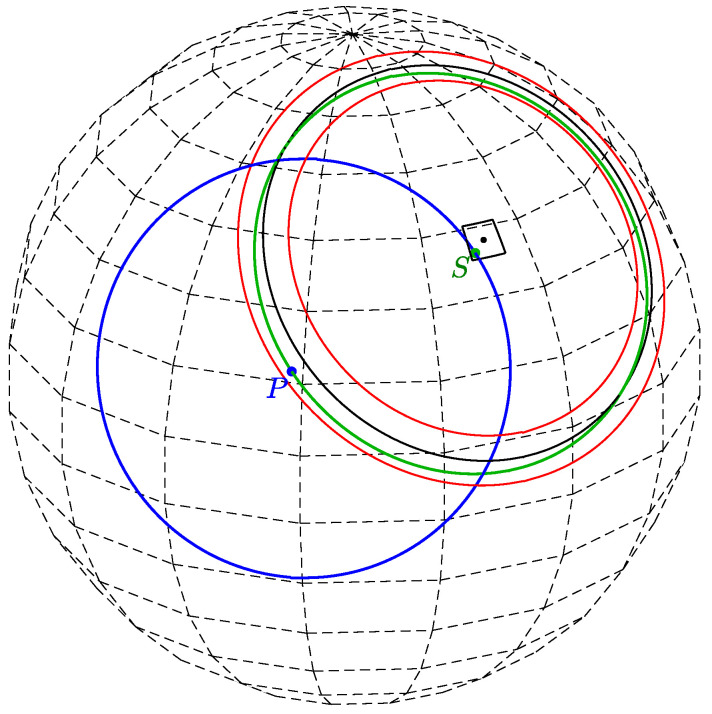
The primary point *P* (blue) with the corresponding primary circle (blue), which votes for the cell with the maximum count. The secondary point *S* on the primary circle and inside the cell defines a secondary circle (green) passing exactly through the primary point *P*. The primary point *P* is located within a spherical segment associated with the cell. The black circle is the common secondary circle defined by the center of the cell, and the red circles are the lower and higher critical circles.

Referring to Figure 14, let *P* be a primary point of which the primary circle (blue point and blue circle) votes for a cell centered at $\left(\theta_{max},\varphi_{max}\right)$. In other words, the primary circle defined by the primary point *P* intersects the cell $\left(\theta_{max},\varphi_{max}\right)$. This implies that the coordinates of any point on the primary circle within the cell, i.e., any secondary point *S* in the cell (green point), are bounded between $\theta =(\theta_{max}-\Delta \theta /2,\theta_{max}+\Delta \theta /2]$ and $\varphi =(\varphi_{max}-\Delta \varphi^{k}/2,\varphi_{max}+\Delta \varphi^{k}/2]$. Consider the common secondary circle defined by the cell center (black circle) and the nearby secondary circle associated with any other secondary point within the cell (green circle). The nearby secondary circle passes through the primary point *P*, whereas the common secondary circle generally passes just near *P*.

**Figure 15:**
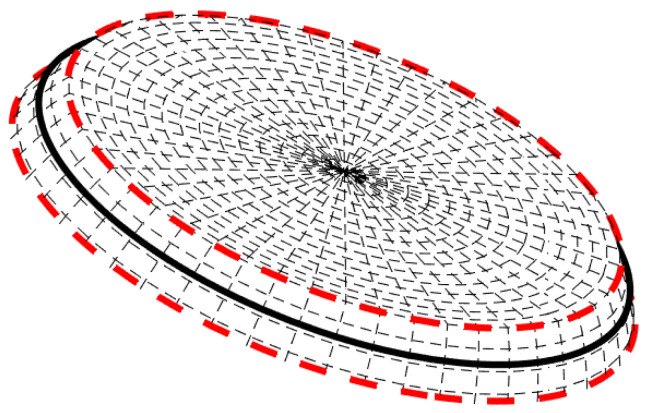
Spherical segment defined by the lower and higher critical circles (red). The black circle is the common secondary circle.

The angle between the radius-vector pointing at the cell center and the radius vector pointing at the nearby secondary point is less than or equal to γ, and equality to γ is achieved when the nearby secondary point coincides with one of the corner points of the cell. The radius vectors are normals of the planes containing these circles. So, the angle between the plane containing the common secondary circle and the plane containing the nearby secondary circle is less than γ. The critical circles are defined such that the angle between the radius vector pointing to a point on the critical circle and the radius vector pointing to its closest point on the common secondary circle is γ. So, the nearby secondary circle lies on a spherical segment (see Figure 15) containing the common secondary circle and delimited by the two critical circles (red circles in Figure 14 and Figure 15). In other words, the angle between the radius vector pointing to the primary point *P* and the radius vector pointing to its closest point on the common secondary circle is less than γ. Hence, the orthodromic distance (great circle distance) of the primary point *P* to each point on the common secondary circle is upper-bounded by R·γ, where *R* is the radius of the sphere.

As shown in Appendix A, γ can be readily expressed in terms of the almost-equal-sized cellquantization parameters, ΔS and Δθ. For a unit sphere

(17) $$\gamma \approx \frac{1}{2}\sqrt{{\left(\Delta \theta \right)}^{2}+{\left(\frac{\Delta S}{\Delta \theta }\right)}^{2}}$$

In this case γ is approximately half of the diagonal of the almost-equal-sized cell. For a sphere of radius *R*, ΔS in Equation (17) should be scaled down by $R^{2}$.

To conclude, the primary point of which the primary circle votes for a cell centered at $\left(\theta_{max},\varphi_{max}\right)$ is located on the spherical segment defined by the cell center. The orthodromic distance between the primary point and the common secondary circle (defined by the cell center) is upper-bounded by R·γ, where *R* is the sphere radius and γ is the central angle between the radius vector $\left(\theta_{max},\varphi_{max}\right)$ and one of the radius vectors $\left(\theta_{max}\pm \Delta \theta /2\;,\;\varphi_{max}\pm \Delta \varphi^{k}/2\right)$ pointing at the cell corners, expressible in terms of the almost-equal-sized cell area.

### 6.4. Improved Algorithm for General (Radius r) Circle Detection on a Sphere

**Figure 16:**
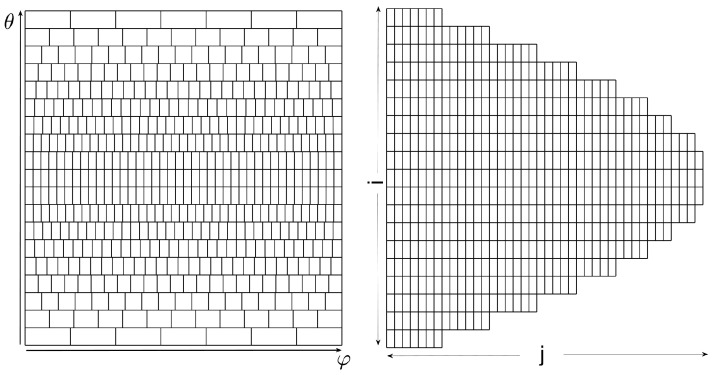
Two illustrations of the accumulator array *M* with almost-equal-sized cell quantization. Left: The accumulators tessellating the θ−φ plane. Right: Accumulators represented by identical rectangles, where the *i* axis represents the nearest integer multiple of Δθ and the *j* axis represents the nearest integer multiple of $\Delta \varphi^{i}$.

Consider the problem of general (any given radius *r*) circle detection on a sphere, as presented in Section 5. We now apply the almost-equal-sized cell quantization scheme, and vote for all cells intersected by the voting curve. For each primary point in the dataset, the algorithm first calculates the limiting values of θ, and loops over all multiples of Δθ between the limiting values $\theta_{lim}^{I,II}$. For each θ, it computes the φ∈[0,2π) coordinates of the points A and B by substituting θ±Δθ/2 in Equation (8). It then increments all the accumulators which correspond to the discrete points with θ and $\varphi \in [\varphi_{A}$, $\varphi_{B}]$. Finally, the accumulator $\left(\theta_{max},\varphi_{max}\right)$ with the maximal reading indicates the spherical circle of radius *r* passing through the largest subset of (primary) points in the data-set. This leads to Algorithm 3:

Note that the accumulator array *M* in this algorithm is no longer a rectangular matrix. The azimuthal step between adjacent accumulators in the $\theta_{i}$ layer is θ-dependent and equals $\Delta \varphi^{i}$. Therefore, the number of accumulators in each $\theta_{i}$ is different. Figure 16 (left) shows the accumulator array in the θ−φ plane. In Figure 16 (right), the accumulators are represented by identical rectangles, where the *i* axis represents the nearest integer multiple of Δθ and the *j* axis represents the nearest integer multiple of $\Delta \varphi^{i}$.
**Algorithm 3:** Improved general circle detection on a sphereInitialize the accumulator array *M* with zeros.Normalize the radius r by the sphere radius R.Calculate *d* by Equation (2).For each primary point 1 to *N*      Calculate $\theta_{lim}^{I,II}\in \left[0,\pi \right)$ by Equation (12).      For each θ=i·Δθ (i∈Z) from $\min\{\theta_{lim}^{I,II}\}$ until $\max\{\theta_{lim}^{I,II}\}$:            For both $\varphi^{I}$ and $\varphi^{II}$ in Equation (8):                  1. Calculate $\Delta \varphi^{i}$ by Equation (14).                  2. Calculate $\varphi_{A}^{I}$ and $\varphi_{B}^{II}$ by substituting $\theta \pm \frac{\Delta \theta }{2}$ in Equation (8).                  3. Choose *n* such that $\varphi_{A}=\varphi_{A}^{I,II}\in \left[0,2\pi \right)$ and $\varphi_{B}=\varphi_{B}^{I,II}\in \left[0,2\pi \right)$                  4. Round $\varphi_{A}$ and $\varphi_{B}$ to the nearest integer multiples of $\Delta \varphi^{i}$.                  5. Increment the accumulators from to $\left(\varphi_{A},\theta \right)$ to $\left(\varphi_{B},\theta \right)$Find $\theta_{max}$ and $\varphi_{max}$ corresponding to the entry with the largest accumulation in *M*.

## 7. Great Circle Examples

What is the great circle on planet Earth that passes through the largest number of airports? Furthermore, what is the great circle that passes through the largest number of cities? We answer these questions using the proposed algorithms, relying on city [24] and airport [25] databases.

### 7.1. Uniform Quantization in the Plane (Algorithm 1) Examples

**Figure 17:**
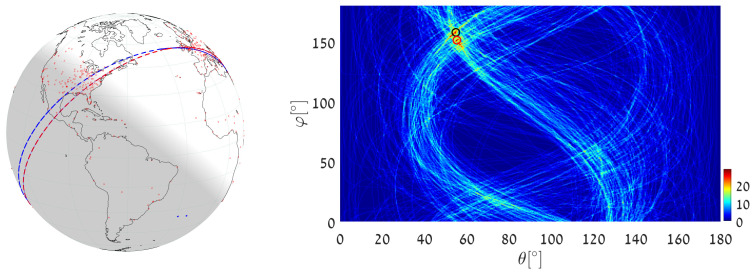
Executing Algorithm 1 on the airport database. (**Left**) The blue great circle passes through the maximum number of airports (red dots). The red great circle corresponds to the second maximum, following the unvoting process. (**Right**) Heat plot of the accumulator array *M*, showing the parameters of the two great circles.

**Figure 18:**
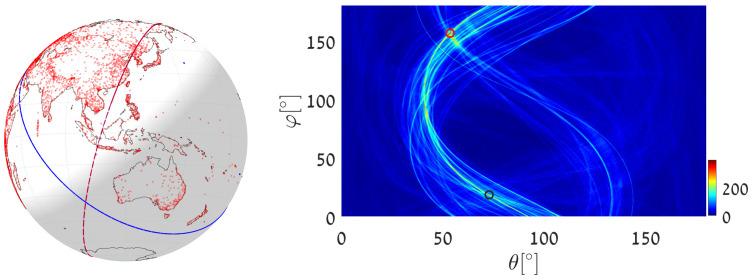
Executing Algorithm 1 on the city database. (**Left**) The blue great circle passes through the maximum number of cities (red dots). The red circle corresponds to the second maximum, following the unvoting process. (**Right**) Heat plot of the accumulator array *M*, showing the parameters of the two great circles.

**Figure 19:**
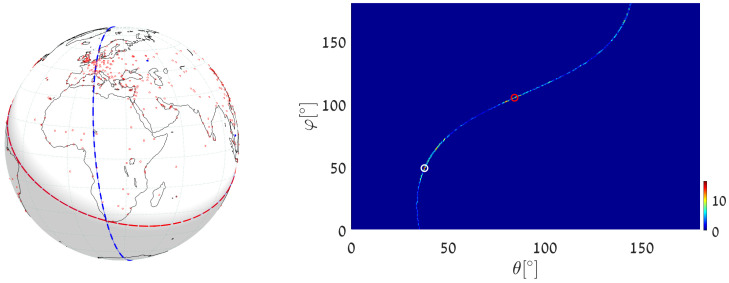
(**Left**) Detecting the great circle (blue) passing through Cape Town International Airport and through the maximum number of other airports, using uniform quantization in the plane. The red circle corresponds to the second maximum, following the unvoting process. (**Right**) Heat plot of the accumulator array *M* along the θ(φ) curve corresponding to Cape Town airport, showing the parameters of the two great circles.

(1) We execute Algorithm 1, taking the world’s major airports (red dots in Figure 17) as input primary points (a total of 617 points). The parameter space is discretized with $\Delta \varphi =\Delta \theta =0.5^{\circ }$. The great circle that passes through the maximum number of airports (blue circle in Figure 17) is on the great circle plane with normal $\theta_{max}=55.25^{\circ },\;\varphi_{max}=151.25^{\circ }$ and visits 29 airports, listed in Appendix B. Note that here and in the following, the number of digits after the decimal point in $\theta_{max}$ and $\varphi_{max}$ relates to the location of the cell center. Obviously, the accuracy of the detected circle parameters follows from the cell size, i.e., from Δθ and Δφ. In order to find the circle passing through the largest number of remaining airports (excluding the 29 airports associated with the first maximum), we identify all primary points of which the voting contributed to the first maximum and remove their votes (unvote [5,6,7]). The new maximum represents the great circle passing through the largest subset of remaining (primary) points in the data-set. It lies on the great circle plane with normal $\theta_{max_{2}}=54.75^{\circ },\;\varphi_{max_{2}}=157.75^{\circ }$ and visits 28 different airports.
(2) We again execute Algorithm 1, this time taking 12,960 world cities (red dots in Figure 18) as input primary points. The parameter space is discretized with $\Delta \varphi =\Delta \theta =0.25^{\circ }$. The great circle (blue circle in Figure 18) that passes through the maximum number of cities is on the great circle plane with normal $\theta_{max}=53.625^{\circ },\varphi_{max}=156.375^{\circ }$ and visits 397 different cities. The great circle that passes through the maximum number of remaining cities (i.e., the maximum after unvoting) is on the great circle plane with normal $\theta_{max_{2}}=72.875^{\circ },\varphi_{max_{2}}=18.375^{\circ }$ and visits 321 cities.
(3) We execute the algorithm in order to find the great circle that passes through a specific airport and the maximum number of other airports. Instead of searching for the maximum value in all the accumulators, we search only in accumulators along the θ(φ) curve corresponding to the specific airport. This reduces the computation time, since we search in $N_{\varphi }$ instead of $N_{\varphi }\cdot N_{\theta }$ accumulators.
  Taking Cape Town International Airport as the specific airport, the great circle that passes through Cape Town International Airport and through the maximum number of other airports is defined by the normal direction $\theta_{max}=84.25^{\circ },\varphi_{max}=104.75^{\circ }$ (blue circle in Figure 19) and visits 16 different airports, listed in Appendix C. In this case, the discretization of the parameter space is $\Delta \varphi =\Delta \theta =0.5^{\circ }$. The great circle that passes through Cape Town International Airport and through the maximum number of remaining airports (the maximum after unvoting, i.e., excluding the airports associated with the first maximum) is on the great circle plane with normal $\theta_{max_{2}}=37.75^{\circ },\varphi_{max_{2}}=48.75^{\circ }$ and visits 13 different airports.

### 7.2. Almost-Equal-Sized Cell Quantization Examples

We now address the same great-circle detection tasks using almost-equal-sized cell quantization. To achieve this, we apply Algorithm 3 (general circle detection) with r=R hence d=0.

**Figure 20:**
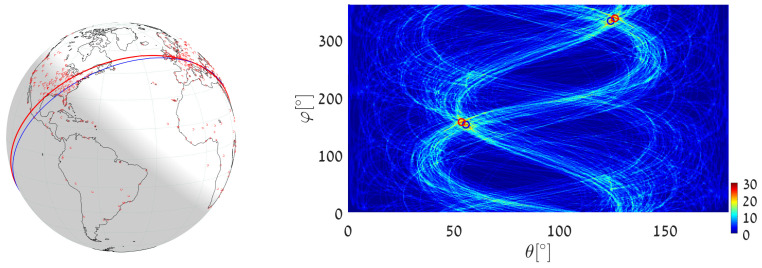
Executing Algorithm 3 on the airport database: (**Left**) The blue great circle passes through the maximum number of airports (red dots). The red great circle corresponds to the second maximum, following the unvoting process. (**Right**) Heat plot of the accumulator array *M*, showing the parameters of the two great circles.

**Figure 21:**
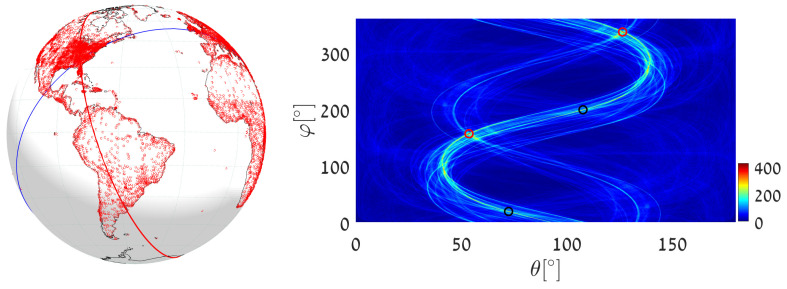
Executing Algorithm 3 on the city database. (**Left**) The blue great circle passes through the maximum number of cities (red dots). The red circle corresponds to the second maximum, following the unvoting process. (**Right**) Heat plot of the accumulator array *M*, showing the parameters of the two great circles.

**Figure 22:**
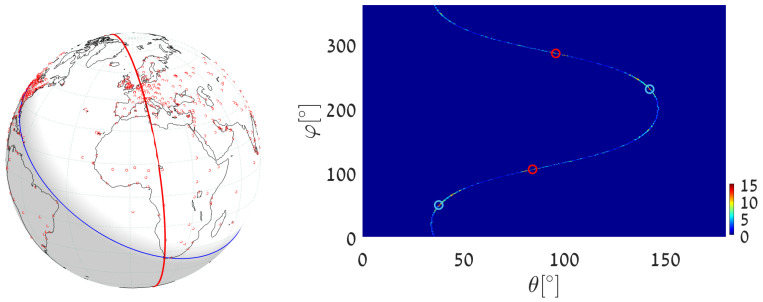
(**Left**) Detecting the great circle (blue) passing through Cape Town International Airport and passing through the maximum number of other airports, using almost-equal-sized cell quantization. The red circle corresponds to the second maximum, following the unvoting process. (**Right**) Heat plot of the accumulator array *M* along the θ(φ) curve corresponding to Cape Town airport, showing the parameters of the two great circles.

(1) We execute Algorithm 3 with d=0, taking the world’s major airports as primary input points. We set $\Delta \theta =0.5^{\circ }$ and $\Delta S=\frac{\pi }{360}\cdot \frac{\pi }{360}=\frac{\pi^{2}}{129,600}\left[{\mathrm{Rad}}^{2}\right]$, which yields accuracy similar to the worst-case accuracy (at θ=π/2) in the above uniform quantization example. The great circle (the blue circle in Figure 20) that passes through the maximum number of airports is on the great circle plane with normal $\theta_{max}=53.75^{\circ }$, $\varphi_{max}=156.4544^{\circ }$. It visits 30 different airports, listed in Appendix D. The great circle that passes through the largest number of remaining airports (the maximum after unvoting) is on the great circle plane with $\theta_{max_{2}}=124.25^{\circ },\varphi_{max_{2}}=331.8655^{\circ }$ and visits 27 airports.
(2) We execute Algorithm 3 with d=0, taking 12,960 world cities (red dots in Figure 18) as input primary points. We set $\Delta \theta =0.25^{\circ }$ and $\Delta S=\frac{\pi }{720}\cdot \frac{\pi }{720}=\frac{\pi^{2}}{518,400}\left[{\mathrm{Rad}}^{2}\right]$. The great circle (the blue circle in Figure 21) that passes through the maximum number of cities is on the great circle plane with normal $\theta_{max}=53.625^{\circ },\varphi_{max}=156.3934^{\circ }$ and visits 425 cities. The great circle that passes through the maximum number of remaining cities (the maximum after unvoting) is on the great circle plane with normal $\theta_{max_{2}}=72.375^{\circ },\varphi_{max_{2}}=18.7609^{\circ }$ and visits 335 cities.
(3) We execute Algorithm 3 with d=0 in order to find the great circle that passes through a specific airport and through the maximum number of other airports. Instead of searching for the maximum value in all the accumulators, we search only in accumulators along the θ(φ) curve corresponding to the specific airport. We again take Cape Town International Airport as the specific airport, now with $\Delta \theta =0.5^{\circ }$ and $\Delta S=\frac{\pi }{360}\cdot \frac{\pi }{360}=\frac{\pi^{2}}{129,600}\left[Rad^{2}\right]$. The great circle (the blue circle in Figure 22) that passes through Cape Town International Airport and through the maximum number of other airports is defined by the normal direction $\theta_{max}=37.75^{\circ },\varphi_{max}=49.3878^{\circ }$, and visits 15 different airports listed in Appendix E. The great circle that passes through the Cape Town International Airport and through the maximum number of remaining airports (the maximum after unvoting, i.e., excluding the airports associated with the first maximum) is on the great circle plane with normal $\theta_{max_{2}}=84.25^{\circ },\varphi_{max_{2}}=104.8324^{\circ }$ and visits 14 different airports.

The number of data points (airports or cities) associated with the great circle detected depends on the size of the parameter-space quantization cells, i.e., on the quantization level. For the examples in this subsection (almost-equal-sized cell quantization), the dependence is presented in Appendix F.

**Figure 23:**
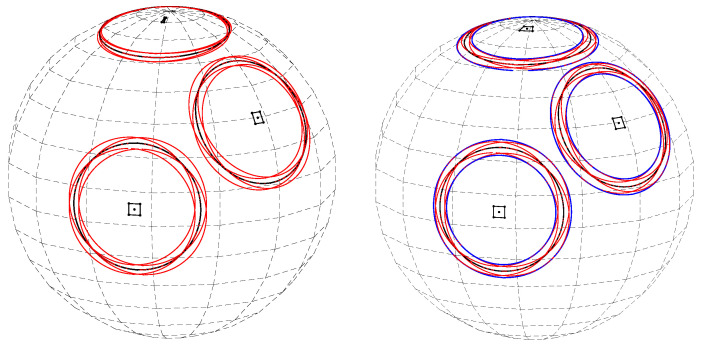
(**Left**) Cells corresponding to uniform quantization in the plane; cells close to the equator are larger than cells close to the poles. Corner circles (red) of cells closer to the pole are closer to each other than corner circles of cells closer to the equator. (**Right**) Almost-equal-sized cell quantization: all cells are of approximately the same size, so the spherical segments between the upper and lower critical circles (blue) cover similar areas.

The differences between the results obtained with uniform quantization in the plane and the those achieved using almost-equal-sized cell quantization are due to the non-equal cell sizes on the sphere yielded by uniform quantization in the plane. Referring to Figure 23, with uniform quantization in the plane (Figure 23 (left)), cells that are close to the equator are larger than cells close to the poles. In contrast, with almost-equal-sized cell quantization (Figure 23 (right)), all cells have approximately the same size. In the above experiments, we chose ΔS such that the smallest $\Delta \varphi_{k}$ in the almost-equal-sized quantization scheme was equal to Δφ used in uniform quantization in the plane. Thus, with almost-equal-sized quantization, the size of the cell on the sphere is larger or equal to that obtained using uniform quantization in the plane. In other words, given cell coordinates $\theta_{max}$ and $\varphi_{max}$ on the sphere, the cell at these coordinates using almost-equal-sized cell quantization is larger or equal in area to the cell at the same coordinates using uniform quantization in the plane. Thus, although the great circle on planet Earth that passes through the largest number of cities is about $\theta_{max}=53.625^{\circ },\varphi_{max}=156.125^{\circ }$ using both quantization schemes, with uniform quantization in the plane it is associated with 397 cities, whereas with almost-equal-sized cell quantization the number of cities is 425.

## 8. General (Small) Circle Examples

In the previous section we found great circles on planet Earth passing through the largest possible number of airports or cities. In this section, we detect small circles of a given radius *r* that visit as many airports or cities as possible. We use algorithm 3 (almost-equal-sized cell quantization). We normalize the radius of the sphere (planet earth) to R=1; hence, 0<r<1.

**Figure 24:**
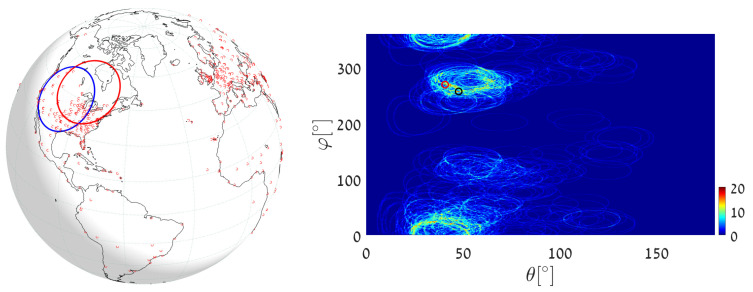
Executing Algorithm 3 with r=0.25 on the airport database. (**Left**) The blue circle passes through the maximum number of airports (red dots). The red circle corresponds to the second maximum, following the unvoting process. (**Right**) Heat plot of the accumulator array *M*, showing the parameters of the two circles.

**Figure 25:**
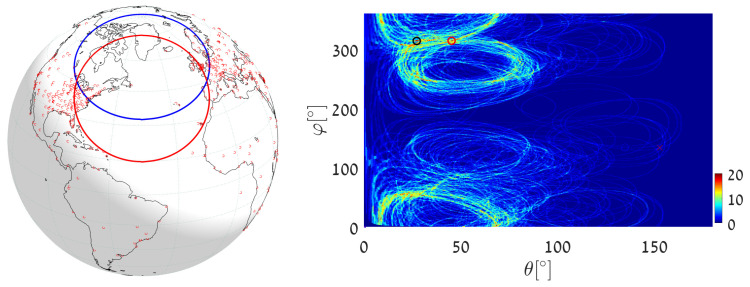
Executing Algorithm 3 with r=0.5 on the airport database. (**Left**) The blue circle passes through the maximum number of airports (red dots). The red circle corresponds to the second maximum, following the unvoting process. (**Right**) Heat plot of the accumulator array *M*, showing the parameters of the two circles.

**Figure 26:**
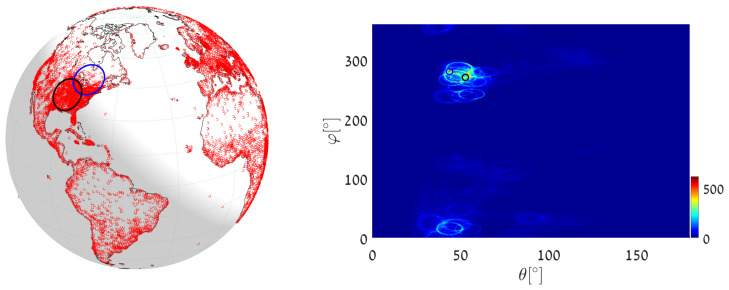
Executing Algorithm 3 with r=0.125 on the city database. (**Left**) The blue circle passes through the maximum number (616) of cities (red dots). The black circle, passing through 386 cities, corresponds to the second maximum, following the unvoting process. (**Right**) Heat plot of the accumulator array *M*, showing the parameters of the two circles.

**Figure 27:**
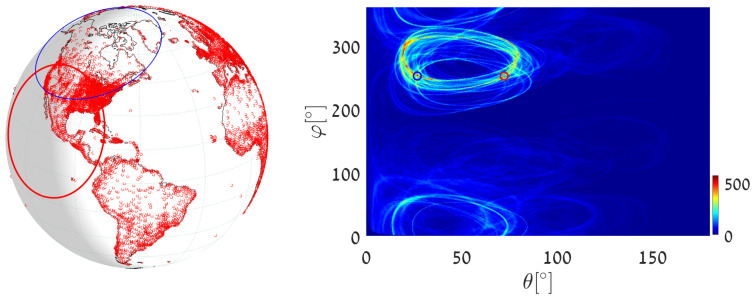
Executing Algorithm 3 with r=0.5 on the city database. (**Left**) The blue circle passes through the maximum number (568) of cities (red dots). The red circle, passing through 523 cities, corresponds to the second maximum, following the unvoting process. (**Right**) Heat plot of the accumulator array *M*, showing the parameters of the two circles.

(1) We execute algorithm 3 with r=0.25 (d≈0.968), taking the world’s largest airports as input primary points. We set $\Delta \theta =0.5^{\circ }$ and $\Delta S=\frac{\pi }{360}\cdot \frac{\pi }{360}=\frac{\pi^{2}}{129,600}\left[{\mathrm{Rad}}^{2}\right]$. The circle of radius r=0.25 that passes through the maximum number of airports (the blue circle in Figure 24) is on the circle plane with normal $\theta_{max}=47.75^{\circ },\varphi_{max}=256.998^{\circ }$ and visits 20 large airports. The circle of radius r=0.25 that passes through the largest number of remaining airports (red circle in Figure 24, corresponding to the maximum after unvoting) is on the circle plane with normal $\theta_{max_{2}}=40.75^{\circ }$, $\varphi_{max_{2}}=269.2340^{\circ }$ and visits 19 different airports.
(2) We now execute algorithm 3 with r=0.5 (d≈0.866), again taking the world’s largest airports as input primary points. We set $\Delta \theta =0.5^{\circ }$ and $\Delta S=\frac{\pi }{360}\cdot \frac{\pi }{360}=\frac{\pi^{2}}{129,600}\left[{\mathrm{Rad}}^{2}\right]$. The circle of radius r=0.5 that passes through the maximum number of airports (the blue circle in Figure 25) is on the circle plane with normal $\theta_{max}=27.25^{\circ }$, $\varphi_{max}=313.6364^{\circ }$ and visits 20 large airports. The circle of radius r=0.5 that passes through the largest number of remaining airports (red circle in Figure 25, corresponding to the maximum after unvoting) is on the circle plane with normal $\theta_{max_{2}}=45.25^{\circ },\varphi_{max_{2}}=313.8552^{\circ }$ and visits 19 different airports.
(3) We execute algorithm 3 with r=0.125 (d≈0.992), this time taking the input primary points from the city database. We set $\Delta \theta =0.5^{\circ }$ and $\Delta S=\frac{\pi }{360}\cdot \frac{\pi }{360}=\frac{\pi^{2}}{129,600}\left[{\mathrm{Rad}}^{2}\right]$. The circle of radius r=0.125 that passes through the maximum number of cities (the blue circle in Figure 26) is on the circle plane with normal $\theta_{max}=43.7500^{\circ }$, $\varphi_{max}=280.1205^{\circ }$ and visits 616 cities. The circle of radius r=0.125 that passes through the largest number of remaining cities (the black circle in Figure 26, corresponding to the maximum after unvoting) is on the circle plane with normal $\theta_{max_{2}}=52.7500^{\circ },\varphi_{max_{2}}=270.4712^{\circ }$ and visits 386 cities.
(4) We execute algorithm 3, now with r=0.5 (d≈0.866), taking the input primary points from the city database. We set $\Delta \theta =0.5^{\circ }$ and $\Delta S=\frac{\pi }{360}\cdot \frac{\pi }{360}=\frac{\pi^{2}}{129,600}\left[{\mathrm{Rad}}^{2}\right]$. The circle of radius r=0.5 that passes through the maximum number of cities (the blue circle in Figure 27) is on the circle plane with normal $\theta_{max}=26.7500^{\circ }$, $\varphi_{max}=251.6667^{\circ }$ and visits 568 cities. The circle of radius r=0.5 that passes through the largest number of remaining cities (the black circle in Figure 26, corresponding to the maximum after unvoting) is on the circle plane with normal $\theta_{max_{2}}=72.2500^{\circ },\varphi_{max_{2}}=251.6327^{\circ }$ and visits 523 cities.

The experimental results in Section 7 and Section 8 were obtained using Matlab on a low-end personal computer with i5-8265U 1.6GHz CPU and 8.00 GB RAM. The Matlab computing time for the various experiments is summarized in Table 1. As expected, computing time was roughly proportional to the number of data points, and increased as the parameter space quantization was refined.

## 9. Conclusions

In this paper we present the first comprehensive solution to the problem of detecting circles, great and small, in a spherical set of points. The suggested approach follows the Hough transform methodology and addresses the fundamental issues associated with Hough algorithms, including parameterization, parameter-space quantization, and analysis of the quantization-induced error. The time-complexity of the proposed algorithms is linear in the number of data-points. The required memory size is simply the number of cells in the accumulator array, which is bounded and two-dimensional in the case of great circles and in the case of small circles of known radius.

Potential applications for this research arise in cases where the data are naturally spherical and where circular features need to be detected. The planetary and geophysical sciences provide numerous examples, including crater and volcano detection. Computer vision applications arise in omnidirectional image analysis, including fisheye image analysis, where spherical image representations are ubiquitous.

The proposed method can be readily extended to the detection of circles of unknown radius in a spherical set of points. The additional unknown can be accommodated either by sequentially applying the known-radius algorithm with different radii, or by adding a dimension representing the unknown radius to the accumulator array.

## Figures and Tables

**Table 1:** Total computing time and computing time per-point (msec) using Matlab on a low-end PC. Results are reported for great-circle detection (r=1) and small-circle detection (r<1) and for different parameter-space resolution values (Δθ and Δφ for Algorithm 1, Δθ and ΔS for Algorithm 3).

Dataset	Algorithm 1				Algorithm 3			
	Δ*θ*	Δ*φ*	Computing Time (ms)		Δ*θ*	Δ*S*	Computing Time (ms)	
			Total Time	Avg. per Point			Total Time	Avg. per Point
Airports	2	2	366	0.43	2	${\left(\frac{\pi }{90}\right)}^{2}$	595	0.93
r=1	1	1	531	0.71	1	${\left(\frac{\pi }{180}\right)}^{2}$	791	1.33
	0.5	0.5	1298	1.98	0.5	${\left(\frac{\pi }{360}\right)}^{2}$	1777	3.02
(616 pts.)	0.25	0.25	3321	5.16	0.25	${\left(\frac{\pi }{720}\right)}^{2}$	3668	5.96
Cities	2	2	3644	0.29	2	${\left(\frac{\pi }{90}\right)}^{2}$	6755	0.53
r=1	1	1	6174	0.47	1	${\left(\frac{\pi }{180}\right)}^{2}$	10,225	0.75
	0.5	0.5	22,779	1.64	0.5	${\left(\frac{\pi }{360}\right)}^{2}$	26,231	2.07
(12,959 pts.)	0.25	0.25	64,385	5.02	0.25	${\left(\frac{\pi }{720}\right)}^{2}$	72,061	5.56
Cities	-	-	-	-	2	${\left(\frac{\pi }{90}\right)}^{2}$	5972	0.48
r=0.5	-	-	-	-	1	${\left(\frac{\pi }{180}\right)}^{2}$	8343	0.70
	-	-	-	-	0.5	${\left(\frac{\pi }{360}\right)}^{2}$	23,403	1.98
(12,959 pts.)	-	-	-	-	0.25	${\left(\frac{\pi }{720}\right)}^{2}$	64,078	4.97
Cities	-	-	-	-	2	${\left(\frac{\pi }{90}\right)}^{2}$	5562	0.43
r=0.125	-	-	-	-	1	${\left(\frac{\pi }{180}\right)}^{2}$	7630	0.57
	-	-	-	-	0.5	${\left(\frac{\pi }{360}\right)}^{2}$	22,423	1.71
(12,959 pts.)	-	-	-	-	0.25	${\left(\frac{\pi }{720}\right)}^{2}$	58,805	4.53

## Data Availability

Not applicable.

## Appendix A. Expressing the Angle *γ* in Terms of Δ*S* and Δ*θ*

In this appendix we express the angle γ between the radius vector pointing at the cell center $\left(\theta_{max},\varphi_{max}\right)$ and that pointing to any of its corners, such as $\left(\theta_{max}+\Delta \theta /2\;,\;\varphi_{max}+\Delta \varphi^{k}/2\right)$.

Assuming a unit sphere, the chord length between the two vector tips is

$$C=\sqrt{{\left(\Delta x\right)}^{2}+{\left(\Delta y\right)}^{2}+{\left(\Delta z\right)}^{2}}$$

where

$$\begin{array}{cc} \Delta x & =\sin\theta \cos\varphi -\sin\left(\theta +\frac{\Delta \theta }{2}\right)\cos\left(\varphi +\frac{\Delta \varphi^{k}}{2}\right)\\ \Delta y & =\sin\theta \sin\varphi -\sin\left(\theta +\frac{\Delta \theta }{2}\right)\sin\left(\varphi +\frac{\Delta \varphi^{k}}{2}\right)\\ \Delta z & =\cos\theta -\cos\left(\theta +\frac{\Delta \theta }{2}\right) \end{array}$$

Applying the first-order Taylor series approximation to $\sin\left(\theta +\frac{\Delta \theta }{2}\right)$ and $\cos\left(\varphi +\frac{\Delta \varphi^{k}}{2}\right)$,

$$\begin{array}{cc} \Delta x & \approx -\frac{\Delta \theta }{2}\cos\theta \cos\varphi +\frac{\Delta \varphi^{k}}{2}\sin\theta \sin\varphi +\frac{\Delta \varphi^{k}\Delta \theta }{4}\cos\theta \sin\varphi \\ \Delta y & \approx -\frac{\Delta \theta }{2}\cos\theta \sin\varphi -\frac{\Delta \varphi^{k}}{2}\sin\theta \cos\varphi +\frac{\Delta \varphi^{k}\Delta \theta }{4}\cos\theta \cos\varphi \\ \Delta z & \approx +\frac{\Delta \theta }{2}\sin\theta \end{array}$$

The third terms in Δx and Δy are negligible (second-order) compared to the other (first-order) terms. Substituting Equation (14),

$$\begin{array}{cc} \Delta x & \approx -\frac{\Delta \theta }{2}\cos\theta \cos\varphi +\frac{\Delta S}{2\Delta \theta }\sin\varphi \\ \Delta y & \approx -\frac{\Delta \theta }{2}\cos\theta \sin\varphi -\frac{\Delta S}{2\Delta \theta }\cos\varphi \\ \Delta z & \approx +\frac{\Delta \theta }{2}\sin\theta \end{array}$$

Squaring, we obtain

$$\begin{array}{cc} {\left(\Delta x\right)}^{2} & \approx \frac{{\left(\Delta \theta \right)}^{2}}{4}\cos^{2}\theta \cos^{2}\varphi +\frac{{\left(\Delta S\right)}^{2}}{4{\left(\Delta \theta \right)}^{2}}\sin^{2}\varphi -\frac{\Delta S}{4}\cos\theta \cos\varphi \sin\varphi \\ {\left(\Delta y\right)}^{2} & \approx \frac{{\left(\Delta \theta \right)}^{2}}{4}\cos^{2}\theta \sin^{2}\varphi +\frac{{\left(\Delta S\right)}^{2}}{4{\left(\Delta \theta \right)}^{2}}\cos^{2}\varphi +\frac{\Delta S}{4}\cos\theta \sin\varphi \cos\varphi \\ {\left(\Delta z\right)}^{2} & \approx \frac{{\left(\Delta \theta \right)}^{2}}{4}\sin^{2}\theta \end{array}$$

Substituting in the definition of *C*,

$$\begin{array}{cc} C & \approx \sqrt{\frac{{\left(\Delta \theta \right)}^{2}}{4}\left(\cos^{2}\theta \left(\cos^{2}\varphi +\sin^{2}\varphi \right)+\sin^{2}\theta \right)+\frac{{\left(\Delta S\right)}^{2}}{4{\left(\Delta \theta \right)}^{2}}\left(\sin^{2}\varphi +\cos^{2}\varphi \right)}\\ & \approx \frac{1}{2}\sqrt{{\left(\Delta \theta \right)}^{2}+{\left(\frac{\Delta S}{\Delta \theta }\right)}^{2}} \end{array}$$

Thus, the central angle γ between the two vectors is

$$\gamma =\arcsin C\approx C\approx \frac{1}{2}\sqrt{{\left(\Delta \theta \right)}^{2}+{\left(\frac{\Delta S}{\Delta \theta }\right)}^{2}}$$

## Appendix B. The 29 Airports Located on the Great Circle That Passes through the Maximum Number of Airports—Uniform Quantization

	**Airport Name**	**Latitude**	**Longitude**	$\Delta \gamma {[}^{O}]$
1	Prishtina International Airport	42.572800	21.035801	0.289202
2	Pierre Elliott Trudeau Airport (Montreal)	45.470600	−73.740799	0.066198
3	Brussels South Charleroi Airport	50.459202	4.453820	0.108124
4	Stuttgart Airport	48.689899	9.221960	0.032867
5	Karlsruhe Baden-Baden Airport	48.779400	8.080500	0.265290
6	Luton Airport (London)	51.874699	−0.368333	0.120182
7	Heathrow Airport (London)	51.470600	−0.461941	0.274561
8	Stansted Airport (London)	51.884998	0.235000	0.276526
9	RAF (Brize Norton)	51.750000	−1.583620	0.282675
10	Findel International Airport (Luxembourg)	49.623333	6.204444	0.116230
11	Ramstein Air Base	49.436901	7.600280	0.153979
12	Buffalo Niagara International Airport	42.940498	−78.732201	0.087453
13	John Glenn Columbus International Airport	39.998001	−82.891899	0.134049
14	Corpus Christi International Airport	27.770399	−97.501198	0.120094
15	Cincinnati Northern Kentucky Airport	39.048801	−84.667801	0.086447
16	Erie International Tom Ridge Field	42.083127	−80.173867	0.101441
17	William P Hobby Airport (Houston)	29.645399	−95.278900	0.141916
18	George Bush Intercontinental Airport (Houston)	29.984400	−95.341400	0.121704
19	Rickenbacker International Airport (Columbus)	39.813801	−82.927803	0.253096
20	Memphis International Airport	35.042400	−89.976700	0.199325
21	Monroe Regional Airport	32.510899	−92.037697	0.292742
22	Greater Rochester International Airport	43.118900	−77.672401	0.256870
23	Louisville International Standiford Field	38.174400	−85.736000	0.020793
24	Paphos International Airport	34.717999	32.485699	0.020407
25	RAF (Akrotiri)	34.590401	32.987900	0.189568
26	Zagreb Airport	45.742901	16.068800	0.085825
27	Joe Punik Airport (Ljubljana)	46.223701	14.457600	0.158587
28	Don Miguel International Airport (Guadalajara)	20.521799	−103.310997	0.288277
29	Queen Alia International Airport (Amman)	31.722601	35.993198	0.086299

## Appendix C. The 16 Airports Located on the Great Circle That Passes through Cape Town and the Maximum Number of Other Airports—Uniform Quantization

	**Airport Name**	**Latitude**	**Longitude**	$\Delta \gamma {[}^{O}]$
1	Cape Town International Airport	−33.964802	18.601700	0.030981
2	Tunis Carthage International Airport	36.851002	10.227200	0.154490
3	Munster Osnabrck Airport	52.134602	7.684830	0.227835
4	Cologne Bonn Airport	50.865898	7.142740	0.310424
5	Dortmund Airport	51.518299	7.612240	0.085782
6	Karlsruhe Baden-Baden Airport	48.779400	8.080500	0.045184
7	Bergen Airport Flesland	60.293400	5.218140	0.307745
8	Stavanger Airport Sola	58.876701	5.637780	0.247560
9	Ramstein Air Base	49.436901	7.600280	0.253077
10	Hosea Kutako Airport (Windhoek)	−22.479900	17.470900	0.305683
11	Ndjili International Airport (Kinshasa)	−4.385750	15.444600	0.250095
12	Malpensa International Airport (Milan)	45.630600	8.728110	0.078630
13	Genoa Cristoforo Colombo Airport (Genova)	44.413300	8.837500	0.177389
14	Milano Linate Airport (Milan)	45.445099	9.276740	0.275585
15	Zurich Airport (Zurich)	47.464699	8.549170	0.067036
16	Hammamet International Airport (Enfidha)	36.075833	10.438611	0.083588

## Appendix D. The 30 Airports Located on the Great Circle That Passes through the Maximum Number of Airports—Almost-Equal-Sized Cell Quantization

	**Airport Name**	**Latitude**	**Longitude**	$\Delta \gamma {[}^{O}]$
1	South Charleroi Airport (Brussels)	50.459202	4.453820	0.154079
2	Munich Airport	48.353802	11.786100	0.267122
3	Stuttgart Airport	48.689899	9.221960	0.199825
4	Gatwick Airport (London)	51.148102	−0.190278	0.226057
5	Heathrow Airport (London)	51.470600	−0.461941	0.025712
6	RAF (Fairford)	51.682201	−1.790030	0.026802
7	RAF (Brize Norton)	51.750000	−1.583620	0.076266
8	Shannon Airport	52.702000	−8.924820	0.141270
9	Findel International Airport (Luxembourg)	49.623333	6.204444	0.177932
10	Ramstein Air Base	49.436901	7.600280	0.022154
11	Joint Base Andrews (Camp Springs)	38.810799	−76.866997	0.272409
12	Hartsfield Jackson Airport (Atlanta)	33.636700	−84.428101	0.047392
13	Asheville Regional Airport	35.436199	−82.541801	0.251562
14	Bradley International Airport (Hartford)	41.938900	−72.683197	0.156189
15	General Edward Logan Airport (Boston)	42.364300	−71.005203	0.253187
16	Thurgood Marshall Airport (Baltimore)	39.175400	−76.668297	0.093830
17	Ronald Reagan Airport (Washington)	38.852100	−77.037697	0.154835
18	Newark Liberty International Airport	40.692501	−74.168701	0.136928
19	Spartanburg International Airport (Greenville)	34.895699	−82.218903	0.321778
20	Washington Dulles International Airport	38.944500	−77.455803	0.126876
21	La Guardia Airport (New York)	40.777199	−73.872597	0.210181
22	Dobbins Air Reserve Base (Marietta)	33.915401	−84.516296	0.296766
23	Montgomery Regional Airport	32.300598	−86.393997	0.280931
24	Mobile Regional Airport	30.691200	−88.242798	0.310154
25	Philadelphia International Airport	39.871899	−75.241096	0.261567
26	Blacksburg Regional Airport (Roanoke)	37.325500	−79.975403	0.225211
27	Sofia Airport	42.696693	23.411436	0.204411
28	Nikola Tesla Airport (Belgrade)	44.818401	20.309099	0.245757
29	King Khaled International Airport (Riyadh)	24.957600	46.698799	0.135812
30	atl	33.137551	−84.375000	0.338159

## Appendix E. The 15 Airports Located on the Same Great Circle That Passes through Cape Town and the Maximum Number of Other Airports—Almost-Equal-Sized Cell Quantization

	**Airport Name**	**Latitude**	**Longitude**	$\Delta \gamma {[}^{O}]$
1	Cape Town International Airport	−33.964802	18.601700	0.317152
2	Albuquerque International Sunport	35.040199	−106.609001	0.225185
3	Fort Worth Alliance Airport	32.987598	−97.318802	0.072202
4	Cannon Air Force Base (Clovis)	34.382801	−103.321999	0.143119
5	Dallas Love Field (Dallas)	32.847099	−96.851799	0.072541
6	Dallas Fort Worth International Airport	32.896801	−97.038002	0.066215
7	Hollywood Airport (Fort Lauderdale)	26.072599	−80.152702	0.147474
8	Meacham International Airport (Fort Worth)	32.819801	−97.362396	0.098141
9	Biloxi International Airport (Gulfport)	30.407301	−89.070099	0.286861
10	Metropolitan Oakland International Airport	37.721298	−122.221001	0.268331
11	San Francisco International Airport	37.618999	−122.375000	0.155643
12	Norman Y. Mineta Airport (San Jose)	37.362598	−121.929001	0.067841
13	Sarasota Bradenton International Airport	27.395399	−82.554398	0.029516
14	Lynden Pindling Airport (Nassau)	25.039000	−77.466202	0.112437
15	Luis Munoz Marin Airport (San Juan)	18.439400	−66.001801	0.061461

## Appendix F. The Dependence of the Number of Points Associated with the Great Circle Detected on the Parameter Space Quantization Level

**Dataset**	**Quantization**		**Number of Points**	**Parameters of the**	
	**Level**		**Associated with the**	**Great Circle Detected**	
	**(Degrees)**		**Great Circle Detected**	**(Degrees)**	
	Δθ	ΔS		$\varphi_{\max}$	$\theta_{\max}$
Cities	2	${\left(\pi /90\right)}^{2}$	1703	156.2500	53
	1	${\left(\pi /180\right)}^{2}$	1177	155.7093	53.5000
	0.5	${\left(\pi /360\right)}^{2}$	729	156.4544	53.7500
	0.25	${\left(\pi /720\right)}^{2}$	425	156.3934	53.625
	0.2	${\left(\pi /900\right)}^{2}$	359	156.4300	53.7000
	0.1	${\left(\pi /1800\right)}^{2}$	198	155.7920	53.2500
	0.05	${\left(\pi /3600\right)}^{2}$	120	145.0215	48.0750
Airports	2	${\left(\pi /90\right)}^{2}$	78	156.2500	53
	1	${\left(\pi /180\right)}^{2}$	50	157.8840	54.5000
	0.5	${\left(\pi /360\right)}^{2}$	30	156.4544	53.7500
	0.25	${\left(\pi /720\right)}^{2}$	19	150.7367	55.1250
	0.166	${\left(\pi /1080\right)}^{2}$	15	154.3911	52.2500
	0.125	${\left(\pi /1440\right)}^{2}$	13	155.7198	53.4375
	0.1	${\left(\pi /1800\right)}^{2}$	12	151.2559	55.3500
	0.05	${\left(\pi /3600\right)}^{2}$	10	154.8403	56.1750
Airports through Cape Town	2	${\left(\pi /90\right)}^{2}$	39	49.3805	39
	1	${\left(\pi /180\right)}^{2}$	24	49.3151	37.5000
	0.5	${\left(\pi /360\right)}^{2}$	15	49.3878	37.7500
	0.25	${\left(\pi /720\right)}^{2}$	10	49.0724	37.8750
